# The sociodemographic characteristics and clinical features of the late-life depression patients: results from the Beijing Anding Hospital mental health big data platform

**DOI:** 10.1186/s12888-022-04339-7

**Published:** 2022-11-02

**Authors:** Xiao Wang, Wenwang Rao, Xueyan Chen, Xinqiao Zhang, Zeng Wang, Xianglin Ma, Qinge Zhang

**Affiliations:** 1 The National Clinical Research Center for Mental Disorders & Beijing Key Laboratory of Mental Disorders & Department of Psychiatry, Capital Medical University& Beijing Anding Hospital, Capital Medical University, 5 Ankang Lane, Dewai Avenue, Xicheng District, Beijing, China; 2 Unit of Psychiatry, Institute of Translational Medicine, Faculty of Health Sciences, University of Macau, Macao SAR, China

**Keywords:** LLD, Outpatient, Antidepressants

## Abstract

**Background:**

The sociodemographic characteristics and clinical features of the Late-life depression (LLD) patients in psychiatric hospitals have not been thoroughly studied in China. This study aimed to explore the psychiatric outpatient attendance of LLD patients at a psychiatric hospital in China, with a subgroup analysis, such as with or without anxiety, gender differences.

**Methods:**

This retrospective study examined outpatients with LLD from January 2013 to August 2019 using data in the Observational Medical Outcomes Partnership Common Data Model (OMOP-CDM) in Beijing Anding Hospital. Age, sex, number of visits, use of drugs and comorbid conditions were extracted from medical records.

**Results:**

In a sample of 47,334 unipolar depression patients, 31,854 (67.30%) were women, and 15,480 (32.70%) were men. The main comorbidities of LDD are generalized anxiety disorder (GAD) (83.62%) and insomnia (74.52%).Among patients with unipolar depression, of which benzodiazepines accounted for the largest proportion (77.77%), Selective serotonin reuptake inhibitors (SSRIs) accounted for 59.00%, a noradrenergic and specific serotonergic antidepressant (NaSSAs) accounted for 36.20%. The average cost of each visit was approximately 646.27 yuan, and the cost of each visit was primarily attributed to Western medicine (22.97%) and Chinese herbal medicine (19.38%). For the cost of outpatient visits, depression comorbid anxiety group had a higher average cost than the non-anxiety group (*p* < 0.05). There are gender differences in outpatient costs, men spend more than women, for western medicine, men spend more than women, for Chinese herbal medicine, women spend more than men (all *p* < 0.05). The utilization rate of SSRIs and benzodiazepines in female patients is significantly higher than that in male patients (*p* < 0.05).

**Conclusion:**

LLD patients are more commonly women than men and more commonly used SSRIs and NaSSAs. Elderly patients with depression often have comorbid generalized anxiety. LLD patients spend most of their visits on medicines, and while the examination costs are lower.

## Introduction

Older adults face special physical and mental health challenges that need to be recognized. Geriatric depression, also known as Late-life depression (LLD) patients, refers to depression with an onset after the age of 60 years [1, 2]. According to population projections, the proportion of the elderly population suffering from severe depression will increase to 8.2% by 2050 [3]. The low recognition of depression is associated with high morbidity and mortality in the elderly population [4]. Depression is one of the most significant causes of emotional suffering in late life and may also be a contributing factor to the morbidity of many medical disorders [5]. In the face of an ageing world population, the complexity of the biopsychosocial aspects of the human ageing process has become evident, which are often associated with physical, psychological and social overload in old age. Depression is common in old age, and its prognosis is poorer than in younger populations [6].

Depression in older adults may be more persistent than depression in early life, often running a chronic, remitting course [7]. Increased mortality from both suicidality and medical illnesses is also an important comorbidity of depressive disorders in later life [8]. The significance of late-life depression is heightened by the fact that there are an increasing number of elderly individuals [9]. Cognitive disorders are frequently found during late-life depression and may represent markers of depression or be a potential risk factor for progression to a minor or major neurocognitive disorder, especially Alzheimer's disease [10], so the first step of treatment for elderly patients with depression such as outpatient treatment is very important, which can reduce the symptoms of depression and the incidence of further cognitive impairment.

A significant proportion of outpatients’ experience depression or depressive symptoms, highlighting the importance of developing effective management strategies for the early identification and treatment of these conditions among outpatients in clinical practice [11]. A large multicenter observational cohort study [12] aimed at determining excess costs of late-life depression from a societal perspective, the results showed that Unadjusted mean costs in a six-month period for depressed individuals (€5031) exceeded those of non-depressed (€2700) by the factor 1.86 and were higher in all health care sectors considered, statistically significant positive excess costs persisted in all formal health care sectors after adjusting for comorbidity and socio-demographics, recognition of depression by the general practitioner's did not moderate the relationship of depression and health care costs. At present, few studies have focused on the Asian population, especially outpatients, depression among older adults is underappreciated both in the community and in general hospitals in Chinese culture. Hence, outpatient visits of elderly depressive patients aroused our concern.

Thus, we conducted this study to investigate the sociodemographic characteristics and clinical features (e.g., the number of visits, drug treatments, comorbid conditions and medical costs) in LLD patients with outpatient based on a big data platform and examined the subgroup analysis, which can enable us to better understand this disease, may provide useful information for the prevention and treatment of LLD, and formulation of intervention measures information.

## Methods

### Setting and design

This retrospective, cross-sectional study was conducted from January 2013 to August 2019. The present study included 47,334 patients aged 60 or older who visited the clinic in the Department of Psychiatry at Beijing Anding Hospital. The patients were diagnosed with unipolar depression (i.e., depressive episodes, recurrent depressive episodes, major depressive episodes with or without psychiatric disorders and depressive episodes with or without somatic symptoms) based on the criteria of the fifth edition of the Diagnostic and Statistical Manual of Mental Disorders (DSM-5). Patients with multiple missing data or important missing data due to incomplete information or invalid outpatient records were excluded. A complete medical history and physical examination were obtained from the patients.

### Statistical analysis

This study was based on data platform in Beijing Anding hospital affiliated to the capital medical university, which was a mental health alliance of the Beijing-Tianjin-Hebei region. The Observational Medical Outcomes Partnership Common Data Model (OMOP CDM), developed by the Observational Health Healthcare Data Science and Informatics Alliance (OHDSI: https://ohdsi.org/), was used to create a database based on clinical diagnosis and treatment data. It has been reported that [13] OMOP CDM is best suited for large, vertical data sharing based on electronic medical records, and 133 different types of healthcare databases are currently using the data model globally. The platform adopts OMOP CDM to standardize the transformation and storage of multi-dimensional clinical data in hospitals. All data collected were analyzed using R Version 4.0.3. Continuous variables are described as the mean and standard deviation. Categorical data are described as frequencies and percentages. T-tests and chi-square tests were used to characterize differences in gender differences, with or without anxiety by configuration. The level of significance was *P* ≤ 0.05.

## Results

### Demographic and clinical data

A total of 47,334 patients were reported, and the number of visits was 396,054. The average number of visits per person was 8.37, with an average of 58.34 days per person. Out of 47,334 patients, 67.3% were female (31,854), and 32.70% were male (15,480). There were 31,817 patients (67.22%) between 60 and 70 years old, 11,097 patients (23.44%) between 70 and 80 years old and 4129 patients (8.72%) between 80 and 90 years old. According to age group, the older groups exhibited a lower percentage of depression.

A total of 12,177 patients were screened for disease course. The time distribution of the disease course was as follows: 5388 patients (44.24%) within 1 year, 2965 patients (24.35) within 1–5 years, 1870 patients (15.36%) within 5–10 years, and 1954 patients (16.05%) greater than 10 years.

A total of 44,448 patients were screened for antidepressant drug treatment, of which benzodiazepines accounted for the largest proportion (77.77%), Selective serotonin reuptake inhibitors (SSRIs) accounted for 59.00%, a noradrenergic and specific serotonergic antidepressant (NaSSAs) accounted for 36.20%, serotonin and norepinephrine reuptake inhibitors (SNRIs) accounted for 18.44%, norepinephrine and dopamine reuptake inhibitors (NDRIs) accounted for 0.70% and antipsychotics accounted for 29.50%.

**Fig. 1:**
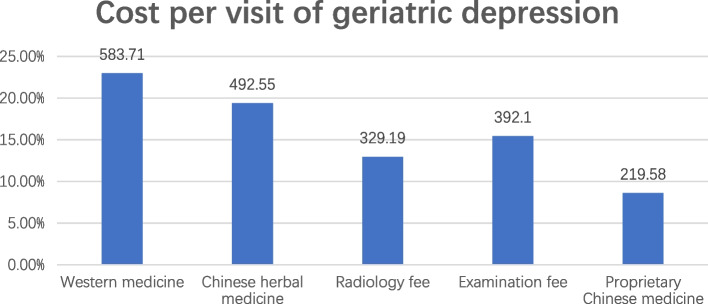
Cost per visit of geriatric depression

The top 3 comorbidities in elderly depressive patients were Generalized anxiety disorder (GAD) (83.62%), insomnia (74.52%) and delusional disorder (16.41%) (Table 1). The average cost of each visit was approximately RMB 646.27 yuan, with most of the costs concentrated in Western medicine (22.97%), Chinese herbal medicine (19.38%) and examination fees (15.43%) (Fig. 1).

**Table 1:** Comorbidity of geriatric depression (*n* = 47,334)

	Person-time	Number of people	Accounted for
Generalized anxiety disorder	311,826	39,580	83.62
insomnia	262,269	35,278	74.52
Delusional disorder	62,955	7767	16.41
Mental disorder	18,107	3654	7.72
Anxiety disorder	10,330	2357	4.98
Obsessional personality disorder	15,605	2110	4.46
Bipolar disorder	17,432	2016	4.26
Dementia	9481	1781	3.76

### Subgroup analysis

#### With or without anxiety

For the cost of outpatient visits, after subgroup analysis, it was found that the depression patients comorbid anxiety group(638.83 ± 402.18) had a higher average cost than the non-anxiety group(554.98 ± 361.15), the difference was statistically significant ( t = 49.03, *p* < 0.05). In terms of specific outpatient expenses, the expenses of patients with depression with anxiety symptoms on western medicine, Chinese herbal medicine, examination fees, and Chinese patent medicine are higher than those without anxiety disorder (all *p* < 0.05), and there is no significant difference in the cost of radiology between the two groups. In terms of antidepressant use, after subgroup analysis, for elderly patients with depression accompanied by anxiety disorders, the proportion of using SSRIs, SNRIs, NaSSAs, antipsychotic drugs, non-benzodiazepine sleep aids and benzodiazepines is higher than that of patients without anxiety disorder (all *p* < 0.05), while there were no significant differences in anti-dementia drugs and NDRI drugs between the two groups.

#### Gender difference

There is a gender difference in the cost of outpatient visits, men(627.43 ± 403.20) spending more than women(619.05 ± 391.94), the difference was statistically significant(t = 5.26, *p* < 0.05). In terms of specific outpatient expenses, men spend more on western medicine than women, and women spend more on Chinese herbal medicine than men, and the differences are statistically significant (all *p* < 0.05). In the radiology department, Chinese patent medicine and examination fees, the gender difference is not obvious. In terms of the use of antidepressants, gender differences still exist, the utilization rate of SSRI and benzodiazepines in female patients is significantly higher than that in male patients, showing statistical differences (all *p* < 0.05), but there was no significant gender difference in NaSSAs,NDRI, antipsychotic drugs, non-benzodiazepine sleep aids, anti-dementia drugs and SNRI drugs.

## Discussion

Major depressive disorder is common later in life and is associated with substantial disability and poor quality of life [14]. Concomitant medical illnesses and depressive symptoms can complicate the LLD, leading to clinically relevant depression that is often overlooked, misunderstood, or even misattributed. Depression in older adults is a mood disorder associated with physical and/or cognitive impairment, it is also different from adult depression in clinical manifestations, drug use, outpatient costs, etc. This study comprehensively describes the comorbidities, socioeconomic status, drug use, and health care consumption of outpatients with depression using a big data platform on mental health and performed a further subgroup analysis.

Our study found that women accounted for more of the depression patients than men in the elderly population, women are about twice as likely as men. Most studies have reported that female is one of the primary predictors of depression later in life [15]. However, there is evidence showing that gender differences in the incidence of depression tend to decrease in extremely old age [16]. Although depression is more common among older women, the gender differences disappear by 80 years of age [17]. The reasons for the above differences may be related to the fact that older women pay more attention to the disease and are more willing to visit the outpatient clinic for treatment.

This study found that among elderly depressive outpatient visits, those aged 60–70 years accounted for the largest proportion, while those aged 80–90 years accounted for a relatively small proportion. Ours study show that depression in the elderly is getting younger and younger, and most are aged 60–70 years, which is also related to the current increase in the incidence of depression in the elderly. The number of depressive patients in China has reached 90 million [18], which requires our further attention. Data have shown that older people are less willing to recognize symptoms of depression or identify as depressed, which may be more pronounced in older age groups [19], which is generally consistent with the results of our study, so for the older population, we should also pay attention to their mental health in time to help them reduce the occurrence of depressive symptoms.

The treatment of LLD is challenging, many older adults do not tolerate or adhere to psychosocial or pharmacologic interventions due to frailty, physical comorbidities, or cognitive impairment. Our study found that antidepressants are more frequently used in outpatient visits of elderly patients with depression, SSRIs and benzodiazepines are more frequently used in women than in men. A meta-analysis conducted by Kok et al. [20] confirmed the benefits of antidepressants in elderly patients. Antidepressants have significant advantages over placebo in terms of remission and/or response [21]. The present study found that SSRIs were still the dominant antidepressant in the elderly population, followed by NaSSAs.The choice of antidepressant is influenced by clinical factors, such as the patient symptoms, medical problems with the condition, current medication intake, the side effects of antidepressants, and in some cases, by practical problems, such as drug costs and insurance coverage. For the elderly population, the expert consensus group recommends that the appropriate treatment plan is citalopram or sertraline at 10–20 mg/d and 25–50 mg/d for elderly depressive patients, respectively [22]. Numerous clinicians believe that mirtazapine, one of the NaSSAs, is very helpful in the treatment of depressed elderly people who suffer from insomnia and loss of appetite [23, 24], and its efficacy is similar to that of amitriptyline or paroxetine, so it is used more in elderly person.

Elderly depression will lead to an increase in the cost of medical care, including increased emergency visits, office visits, increased drug use, higher risk for alcohol and substance use, and increased length of inpatient stay are impacts on society [25]. Our study found that elderly individuals with depression spent approximately 646.27 yuan per visit on average, Western medicine and Chinese herbal medicine were the primary sources of expenditure, and men spend more than women. It is estimated that the medical costs of depressed older adults are about twice or more than those of non-depressed older adults, and the cost of informal medical care is about four times higher [26, 27], depression in the elderly population is associated with a significant increase in medical expenses. One study revealed that even after adjusting for chronic diseases, the total outpatient cost for depressive patients was 43%-52% higher than that of nondepressed elderly patients [28]. Another study conducted in the United States found that the per capita medical expenditure for adult depression in 2005 was estimated to be $173.3 billion, however, the figure was $681 billion in 2010, with the fastest growth rate in the elderly population [29]. In addition, a previous study showed that the average annual medical expenditure per capita caused by depression was approximately $42.67 billion in China [30]. Studies have shown that medical expenditures caused by depression do exist in China, and measures must be taken to improve the mental health of rural elderly people to reduce medical expenditures [31]. In this study, we found that the examination cost of elderly patients with depression is extremely low, which may be related to factors such as the patient's older age, inconvenience in mobility, and unwillingness to come to the outpatient clinic for follow-up visits, most of the drugs are taken by family members. Furthermore, given that elderly patients have a higher incidence of physical diseases, blood drug concentrations and reduced liver and kidney function, clinicians should be regularly monitoring these patients in clinical practice. LLD has high costs for individuals and society, should be paid attention to by relevant social departments, such as a certain preference for the elderly in terms of medical insurance policies to improve depressive symptoms.

This study found that GAD and insomnia are the most common diseases associated with depression in elderly individuals, and patients with anxiety symptoms had higher outpatient costs than those without anxiety symptoms. Depression and anxiety are considerable public health problems for the elderly population. GAD is the most common anxiety disorder in primary care [32, 33]. A previous clinical study found that approximately 65% of older people with depression suffered from anxiety [34]. The top two prevalence rates of anxiety in late-life depression were GAD and agoraphobia [35]. Major depressive disorder (MDD) has the strongest correlation with GAD, while MDD has the weakest correlation with specific phobias [36]. Determinants of comorbid anxiety disorder in elderly depression included young age, female sex, low education level, severe depression, and early trauma [37], we should intervene as soon as possible for elderly depression patients with the above conditions to reduce the incidence of comorbidities. GAD significantly increases the burden of depression due to its impact on quality of life, physical disability, rising use of medical care and mortality [37], higher levels of suicidality [38], and reduced cognitive ability [39] in elderly patients, which was similar to the results of this study. Elderly people with anxiety are mostly accompanied by physical symptoms and are extremely worried about their health [19], they will carry out corresponding outpatient examinations and treatment, which will further increase the cost of outpatient services. The geriatric population is vulnerable with medical comorbidities and unique social situations that can lead to under-treatment and increased costs [40]. It has been confirmed that depression in the elderly is often accompanied by anxiety symptoms, which will increase the outpatient costs of patients, therefore, we need to pay further attention to this population and reduce the incidence of comorbidities of geriatric depression.

This study found that patients with a disease course less than 1 year were the majority, while patients with a disease course of 5–10 years were in the minority, suggesting that new onset of depression in elderly individuals is more common. In a prospective cohort [41], we observed that poor physical health, unhealthy behaviors, and social stressors were significantly associated with an increased risk of depression in the elderly population. Another study reported that poor financial status and physical health, unfilial children, and constant self-perception were important predictors of depression in an elderly population in Beijing [42]. Based on the uniqueness of older patients, they are more prone to depression and have an increased proportion of new onset depression. Socioeconomic status, an important indicator of social structures, has consistently been found to be associated with geriatric depression, a cross-sectional study evaluated the impact of socioeconomic status and housing conditions in geriatric depression in rural China, the results showed that lowest personal annual income, polluting cooking fuel, toilet without seat and having no bath facility were significantly associated with more depressive symptoms [43]. Given that most studies have used the 60-year threshold, early- and late-onset depression were classified in this study based on the same criterion [44]. Patients with early-onset depression have more frequent and longer episodes, according to the glucocorticoid cascade hypothesis [45, 46]. Depressive symptoms are similar throughout the course of illness when depressive disorders occur in early life [47]. Elderly patients with late-onset depression are more severe, and their memory, language fluency and other cognitive abilities are also more likely to be affected [48]. Late‐onset depression may be associated with more serious executive impairment than early‐onset depression [49]. Depression in later life is thought to be a risk factor for cognitive decline, particularly for Alzheimer's disease and vascular dementia [50, 51]. In the future, we will further explore the differences between early-onset depression and late-onset depression in this population, and further clarify their specific types in outpatient care, because it may have a potential impact on the treatment and prognosis of geriatric depression.

There are several limitations associated with our study. Firstly, the information of the outpatient department is not complete enough, the information collected is mostly limited to age, gender, drugs, etc., and the outpatient system needs to be further improved in the future. Second, this study is a single-centre study and only includes data from Beijing Anding Hospital. It is hoped that a multi-centre study will be carried out based on the big data platform of Beijing-Tianjin-Hebei region in the future. Further, the study only discusses geriatric depression, does not include people with dementia, bipolar disorder, etc., which is relatively single and will be further explored in the future.

In conclusion, this study exhaustively described the comorbidities, socioeconomic status, drug use, and health care consumption of outpatients with older depression in the Beijing Anding Hospital Mental Health Big Data Platform. Most elderly depressive patients were female. The top two antidepressants and comorbidities were SSRIs/NaSSAs and anxiety/insomnia, respectively, and there were more new-onset depression cases in this population. Patients with anxiety symptoms have higher outpatient costs and a higher disease burden. Further research on depression in elderly patients is needed to better treat these patients in outpatient clinics.

## Data Availability

The data used in this study are available from the corresponding author upon reasonable request.

## Abbreviations

LLD: Late-life depression
GAD: Generalized anxiety disorder
MDD: Major depressive disorder
OMOP-CDM: Observational Medical Outcomes Partnership Common Data Model
SSRIs: Selective serotonin reuptake inhibitors
NaSSAs: Noradrenergic and specific serotonergic antidepressant
SNRIs: Serotonin and norepinephrine reuptake inhibitors
NDRIs: Norepinephrine and dopamine reuptake inhibitors
DSM-5: The fifth edition of the Diagnostic and Statistical Manual of Mental Disorders
